# PW02-005 - A web registry of genotype-phenotype correlation

**DOI:** 10.1186/1546-0096-11-S1-A145

**Published:** 2013-11-08

**Authors:** M Doglio, R Papa, R Caorsi, S Federici, M Finetti, A Naselli, N Ruperto, A Martini, I Ceccherini, M Gattorno

**Affiliations:** 1 Pediatria 2, IRCCS Istituto Giannina Gaslini, Genoa, Italy; 2 Laboratorio di Genetica Molecolare, IRCCS Istituto Giannina Gaslini, Genoa, Italy

## Introduction

The possible range of clinical manifestations associated to the different mutations associated to autoinflammatory disorders is still largely unknown. A registry of hereditary auto-inflammatory disorders mutations is available on the web (Infevers, http://fmf.igh.cnrs.fr/ISSAID/infevers/). This registry gathers updated information on all mutations responsible for hereditary inflammatory disorders. The clinical phenotype associated with the single mutation in the first reported case is also available.

## Objectives

To provide a web page with the description of the genotype-phenotype correlation found in all the patients enrolled in an international registry for Autoinflammatory diseases (EUROFEVER).

## Methods

For each disease, we created a table describing the correlation between genotype and phenotype in all the patients enrolled in the EUROFEVER registry. In autosomal dominant diseases (CAPS, TRAPS and PAPA syndrome) all mutations were analyzed individually. In autosomal recessive diseases (FMF and MKD), the clinical phenotype of homozygous patients was described. For patients with compound heterozygosis the description of all possible combinations is given. For each mutation, the following items are shown: i) number of patients described, ii) mean age of onset; iii) disease course (recurrent or chronic); iv) prevalent clinical manifestations and duration of fever episodes; v) atypical manifestations; vi) response to treatment; vii) complications.

## Results

We analyzed the genotype-phenotype correlation of 666 patients (313 FMF, 108 CAPS, 72 MKD, 158 TRAPS and 15 PAPA) enrolled in the registry and validated. A summary of the main clinical features associated to 48 variants of *TNFRFS1A*, 33 variants of *MVK* (with 35 combinations for compound heterozygous); 25 variants of *NLRP3*, 16 variants of *MEFV* (with 42 combinations for compound heterozygous) and 6 variants of *PSPTPI1* was performed. For each disease a table with all variable described in method section has been established. A dedicated webpage is on construction in the EUROFEVER web-site (http://www.printo.it/eurofever).

## Conclusion

We provide a useful tool for all the clinicians, creating a web page for the consultation of the correlation between genotype and phenotype in autoinflammatory diseases based on the patients enrolled in the Eurofever registry. This tool is complementary to Infevers database and will be implemented in parallel with the registry.

## Disclosure of interest

None declared.

